# A Perspective on Building Ethical Datasets for Children's Conversational Agents

**DOI:** 10.3389/frai.2021.637532

**Published:** 2021-05-13

**Authors:** Jakki O. Bailey, Barkha Patel, Danna Gurari

**Affiliations:** School of Information, University of Texas at Austin, Austin, TX, United States

**Keywords:** conversational agents, artificial intelligence, children, ethic, datasets

## Abstract

Artificial intelligence (AI)-powered technologies are becoming an integral part of youth's environments, impacting how they socialize and learn. Children (12 years of age and younger) often interact with AI through conversational agents (e.g., Siri and Alexa) that they speak with to receive information about the world. Conversational agents can mimic human social interactions, and it is important to develop socially intelligent agents appropriate for younger populations. Yet it is often unclear what data are curated to power many of these systems. This article applies a sociocultural developmental approach to examine child-centric intelligent conversational agents, including an overview of how children's development influences their social learning in the world and how that relates to AI. Examples are presented that reflect potential data types available for training AI models to generate children's conversational agents' speech. The ethical implications for building different datasets and training models using them are discussed as well as future directions for the use of social AI-driven technology for children.

## Introduction

Children use social interactions as a source of information to understand the world, and artificial intelligence (AI) is playing a greater role in those interactions. For example, in a 2019 U.S. survey, 31% of 8–12-year-olds reported having access to a conversational system home device powered by AI (Rideout and Robb, 2019); and from 2018 to 2019, the UK saw a nearly 2-fold increase in children's access to these devices (Mediacom, 2018). Conversational systems like Alexa, Google Home, or Siri use AI to imitate human responses by both understanding and producing speech or textual responses. Their responses are so lifelike that parents have wondered if their children should be polite to these devices (Rosenwald, 2017, March 2; Baig, 2019). Yet it is unclear what data are being used to train the AI models.

To aid in the process of designing child-centric AI systems, this paper applies a sociocultural developmental approach to examining conversational agents designed for children. For the purposes of this paper, we define children to include early adolescents: those 12 years of age and younger. Our main argument is that conversational agents can act as social peers that children can learn from. We summarize approaches for building large-scale databases that represent conversational speech for children's agents, including a discussion of their strengths, weaknesses, and ethical implications. Finally, we present key next steps for incorporating conversational agents in the lives of children while maintaining their health and safety.

## Learning Through Social Interactions: an Opportunity for Conversational Agents

Human development is a complex system that incorporates many factors across different time scales (Smith and Thelen, 2003; Fox et al., 2010). People's skills and abilities are a combination of genetics, the environment that interacts with those genes (whether the genetic material reaches its full potential), and their experiences in the world (Fox et al., 2010). A sociocultural approach to human development contends that children's interactions in their communities can shape their attitudes and perceptions. According to Rogoff (1990), children are “cognitive apprentices” that learn through observing and participating with others. Children are novices that interact with experts in their communities to develop specific skills. Through these interactions, children discover what ideas and approaches are valued. Experts in a community are not limited to adults but also include children's social peers.

Social conversational agents may shape how youth learn in the future by acting as expert peers. Conversational agents can provide full human-like conversation or provide simple direct responses such as with a recommender system. Artificial conversational agents act behaviorally real by providing social contingency such as “listening,” pausing, and responding to users' verbal requests, and they come in a variety of forms. For instance, AI-powered agents can be embodied (i.e., virtual character and anthropomorphized robot) or simply be a voice emitting from a speaker. Advancements in AI design have allowed artificial agents to provide socially rich conversations, similar to people (Ryokai et al., 2003; Kory-Westlund and Breazeal, 2019; Garg and Sengupta, 2020), and can even respond to users' emotional states (D'mello and Kory, 2015). Because of conversational agents' social realism, children are known to use the same communication strategies with voice-driven characters as in face-to-face conversations (Cheng et al., 2018) and be more motivated to learn with an agent peer than without one (Chase et al., 2009; Biswas et al., 2010; Kory-Westlund and Breazeal, 2019). Furthermore, children can develop deep one-way emotionally tinged relationships with media figures or characters that they view as friends or authority figures (Brunick et al., 2016; Gleason et al., 2017). Social–emotional connections with AI-powered agents can boost learning (Ryokai et al., 2003; Kory-Westlund et al., 2017; Calvert et al., 2020), and the types of emotions that youth experience when interacting with agents can both positively and negatively affect learning outcomes, such as boredom-reducing learning (Baker et al., 2010).

## Generating Data for Children's Conversational Agents

Well-designed AI can enhance the lives of children, and this begins with the data used to run the technology. Typically, machine learning algorithms power social conversational agents. Sutskever et al. (2014) proposed using a sequence-to-sequence method, such as various types of recurrent neural network (RNN) language models (e.g., long short-term memory) for language and conversations. In an RNN model, a neuron at each level or layer stores the state of the previous input while processing the current input. This type of modeling has shown to be efficient in tasks like learning conversation (Sutskever et al., 2014; Vinyals and Le, 2015). More specifically, in a sequence-to-sequence framework, the input sequence is a conversational phrase from the child, and the output is the sequence of an answer to be delivered by the agent. The AI models are typically trained on dialogue databases.

The choices that are made about the content of dialogue databases have the potential to influence children's social learning, including how they view themselves, communicate, and think about other people. For conversational agents to know when and how to provide the appropriate output or response, the model (i.e., RNN) ideally needs to be trained on large amounts of data (Faruqui et al., 2015). Furthermore, the datasets need to be indicative of how youth converse with one another. Research has shown that children prefer conversational agents that mimic children's, not adult's, communication styles (Druga et al., 2017). It is important to note that large datasets and machine learning algorithms are not necessary for effective natural language conversations to socially engage users (Cai et al., 2011). For example, algorithms less sophisticated than RNN models can affect emotions and promote learning (Cai et al., 2011; Forsyth et al., 2015). However, these approaches can limit the generalizability of the agents' conversations beyond a specific domain (e.g., Forsyth et al., 2019).

The dataset leveraged to train the agent can either be built on a preexisting dataset or from newly created data; both options have advantages and disadvantages. Preexisting datasets that have a previous history of use can reduce the amount of time, compared with creating a new dataset, to train neural networks to learn the appropriate set of rules it will use to make decisions. However, using an already created dataset reduces the flexibility to change the content. Curating a new dataset allows the dialogue to be personalized to meet the goals of the project by providing greater ease in manipulating, tweaking, and molding the data than a preexisting dataset. For example, with a newly created dataset, the personality and tone of the agent can be developed for specific use cases and provide opportunities to adjust for bias in the data. However, creating a completely new dataset is a painstakingly time-intensive practice. Creating a new dataset requires identifying the appropriate dialogue and gathering the exact information from that conversation. Regardless of what source is used, the data that are curated will need to account for the specific ranges and domains (e.g., tutoring and social recommendation systems). For example, a conversational agent tutoring a preschooler will be different than a social recommendation system for an 11-year-old.

We present three examples, books, films, and children's real-world interactions, for generating data for child-centered conversational agents that could mimic a social peer. These exemplify ways that designers and researchers can collect data and develop corpora for children's conversational agents. Books and films represent data sources that provide readily available content aimed at child audiences. Books and films are crafted to meet the specific language abilities of specific ages and often contain conversations, themes, and contexts focused on youth. In addition, these longer formats were chosen, as opposed to their shorter counterparts (e.g., short stories and show episodes), because they often contain multiple conversations with continuity around a theme or an idea. Finally, recording youth's real-world conversations could power artificial agents to closely mimic children's own real-world peers. We discuss the benefits, challenges, and ethical implications. An overview of exemplar datasets we discuss is shown in Table 1.

**Table 1:** Comparison of example data sources for children's conversational agents.

**Data source**	**Example references**	**Advantages**	**Disadvantages**
Books	Children's Book Test (Hill et al., 2016)	• Easy to collect • Much data available • Available for social interactions and conversations at different times in history	• Majority of content written as narrative to be read, not spoken conversation • Interpretation of children's conversations by adult content creators • May lack nuanced contemporary speech patterns
Films	MovieQA (Tapaswi et al., 2016)	• Easy to collect • Available for social interactions and conversations at different times in history • Content meant to be spoken conversation	• Can perpetuate negative stereotypes from the film industry • Interpretation of children's conversations by adult content creators
Real-world interactions	Curiosity-evoking virtual agent (Paranjape et al., 2018)	• Available for social interactions and conversations at different times in history • Reflects children's actual speech in social interactions • Flexibility to include diverse representation • Control over tone and purpose • Content meant to be spoken conversation	• Painstakingly time-intensive • Potentially costly • Pronunciation challenges of young populations

### Example 1: Books for Children

Books created for children have the potential to be sourced for training an agent that speaks to and understands children. Books written for young audiences use words and sentence structures that are comprehensible to children, and they often focus on stories and contexts relatable to them. These types of books have become numerous and widely accessible. Several pieces of conversational data around a theme or an idea could potentially support the creation of a large variety of responses to a domain.

Already, datasets have been developed using children's books. For instance, the “Children's Book Test” (CBT) dataset, which represents one of the freely available children's dialogue datasets (Hill et al., 2016), consists of text from 108 children's books. It was made available by Project Gutenberg (“Project Gutenberg” 2020). The CBT was built with the intention of using linguistic models to examine how memory and context influence the way language is processed and understood (Hill et al., 2016).

While children's books can be well-written, they may not always reflect how current language is used to convey social meaning. Language has many facets and is a cultural creation that changes with time. Furthermore, to be most effective, datasets need to be built around an intended use. For example, the CBT dataset is used to test linguistic context, not specifically to train an AI for spoken conversations with children (Hill et al., 2016). In addition, the books used for the CBT were published from 1820 to 1922, with 46% of the books published before 1900 (Hill et al., 2016). The social meaning in language used in the 19th and early 20th centuries is likely to be very different than in language today. Furthermore, themes and harmful outdated cultural norms can persist within older texts. Finally, while text from books may be readily available in large quantities, and datasets like the CBT exist, books are less likely to serve as a valid source for conversational agents that mimic youth's social interactions with peers. For example, texts for older children and early adolescents are likely to contain more text describing a scene or the internal motivations of the characters than a dialogue between them. However, books written for children could act as a supplement or as a starting point for the creation of datasets, but conversational agents built entirely from the text in books will be limited.

### Example 2: Movies for Children

The dialogue in children's films could provide large amounts of raw material for training the AI model. Several films geared toward child audiences are released each year, providing ample examples of social interactions and conversations that progress over time. These movies tend to use language understandable by children and mimic their everyday speech, often reflecting current cultural and social meaning. Unlike books, the dialogue and language in films are meant to be heard not read, potentially demonstrating more nuanced language patterns spoken between peers.

Although there have been a series of databases that are built from movies (Tapaswi et al., 2016; Chu and Roy, 2017; Pecune et al., 2019), few, if any, have been created to train children's conversational agents. For example, MovieQA (Tapaswi et al., 2016) was created to answer questions about the content of movies by using ~15,000 multiple choice question answers, but not to engage in social conversations.

Films can provide ample dialogue between children; however, the film and television industry have specific biases about how cultures, gender identities, languages, class, race, and ethnicity are represented. Studies show that popular and accessible television or film content reinforces stereotypes, normalizing harmful representations of people with regards their race or ethnicity (Ward, 2004; Tahmahkera, 2008; Ramasubramanian, 2011; Tukachinsky, 2015), as well as their gender (Grabe et al., 2008; Harriger et al., 2018). Children's film and television media is no exception. For instance, a content analysis of the top 25 grossing children's films from 2004 to 2016 revealed that over two-thirds of the films emphasized gendered appearances of female as thin and male as muscular (Harriger et al., 2018)—reinforcing body stereotypes that girls or women are not muscular and that all boys and men are highly muscular. Emphasizing stereotypical representations can negatively affect how children as young as 3 years of age view themselves (Grabe et al., 2008; Harriger et al., 2010). Importantly, media stereotypes can become integrated into the types of language that people use in society (e.g., Bligh et al., 2012), infiltrating day-to-day conversations. Dialogue from children's digital media must be carefully selected to avoid perpetuating harmful representations that children could internalize.

### Example 3: Children's Real-World Interactions

An ideal way to build a dataset for conversational agents is to record conversations from children's real-life social interactions (e.g., Paranjape et al., 2018). As mentioned previously, creating a new database provides control over what the agent learns and increases flexibility to mold the dataset to create a child-centric agent. When done well, capturing children's conversations can create culturally and linguistically diverse datasets, beyond the stereotypes or limited views of popular media. It could be argued that this approach reflects a clearer reality of how children speak and behave than in books or films. A database based on children's interactions can capture what they say to each other in the moment as opposed to adults' interpretations of how children think or behave (e.g., dialogue in books or films). Furthermore, the entire dataset can be based on continuous conversations between children with little adult interference, putting an emphasis on peer-to-peer guided participation (i.e., Rogoff, 1990). As such, conversational agents built around youth's real-world interactions will likely mimic a social peer and may encourage children to quickly trust the system. Finally, building new datasets from children's conversations is an important research initiative that further expands the AI community and increases the availability of age-appropriate technology.

While creating a dataset using children's social interactions may be preferable, it is a painstaking and time-intensive process that requires clear and specific planning. Understanding the social meaning of children's conversations involves both the content and sentiment of the words, as well as their associated non-verbal behaviors (Oertel et al., 2013). In addition, it will be important to consider which ages to collect dialogue data from. Databases will need to utilize a basic level of speech proficiency for the speech of the conversational agents. For, example, young children's developing speech patterns may impact their ability to pronounce words. Research has shown conversational agents such as Echo Dot or Alexa can have problems understanding children's speech (Cheng et al., 2018; Beneteau et al., 2019). However, the developing speech patterns of younger children (i.e., the ability to pronounce certain sounds) could instead be used to help the AI system recognize the queries of children still developing more mature speech patterns. This approach could also be applied for systems responding to multilingual children that speak to systems using their non-native language and have variations in pronunciation.

To collect data, at least two children must be brought together to talk in a naturalistic setting where they can interact with one another, such as playing games or learning a lesson (Paranjape et al., 2018). The aim is not to control children's interactions but to facilitate sessions in which they can talk and socially engage with one another. Recording children's responses in a naturalistic setting generates data that includes spontaneous conversational interactions (Oertel et al., 2013). Researchers and designers can determine if bringing pairs of friends, acquaintances, or children that are strangers is appropriate for their project.

Having a diverse set of children from a variety of backgrounds is crucial to the success of building inclusive datasets based on real-world interactions. Creating datasets on social interactions and dialogue originating from the real life of modern children provides a unique opportunity to create a conversational agent that understands and responds to children as a peer. Consideration must be taken into which children are selected for these recorded sessions. Designers and researchers must resist only selecting groups that they would consider “normal” or “typical” because the children share their cultural identity and background or because they live conveniently nearby. By selecting a narrow part of the population, designers inevitably normalize that culture to the potential exclusion and detriment of other children, especially for those from already marginalized communities.

Furthermore, privacy is a vital aspect to consider when building databases from children's conversations. Parents and children must know exactly what information they agree to provide and when, for example, what social interactions and conversations will be recorded and stored for what purposes. While continually tracking and recording children's conversations generates more data to train the model (e.g., in the home), it raises ethical issues with collecting data from minors. For instance, the Council of Parent Attorneys and Advocates (COPAA) contains legal requirements for children's media, typically with applications and websites, that have limitations on what personal information can be tracked and collected (Montgomery et al., 2017). Unfortunately, companies often violate these rules through a variety of loopholes (Montgomery et al., 2017; Reyes et al., 2018). Oversight must be brought in to protect the identities of children and their communities.

## Discussion: Next Steps for the Ethical Design of Children's Social Artificial Intelligence

There are several uses for conversational agents in children's day-to-day lives. For example, they could be a comfort and a source of entertainment for bedridden children in hospitals that have limited mobility and provide a more sanitary option by being touchless. With these opportunities to enhance children's lives, conversational agents need to be designed carefully and ethically. While it is challenging to predict how a technology will transform a society, the safety and socio-emotional well-being of children must be considered.

### Maintaining Privacy

Design based on child development and social interactions will need to incorporate safeguards within the systems for children, particularly for those still developing advanced spoken language skills. One mispronounced word could send a private conversation to an unintended recipient or a stranger. For example, one conversational home device was found to have recorded conversations unbeknownst to the people in the room and then sent the recording to someone else (Shaban, 2018). The system mistakenly heard a sequence of words in a conversation that sounded like a request to record and then sent the data. Public policymakers and researchers will need to investigate how technological enhancements in AI can grow, while still ensuring children's privacy.

### Understanding the Effect of Consumerism on Identity

While conversational agents have the potential to connect children to a plethora of resources and interesting experiences, it is important to remember that these are commercially designed technologies connected to larger companies selling and promoting products. Slick marketing techniques that blur commercial content into the main content are often integrated into children's media experiences (for an extended discussion, see Calvert, 2008). Incentivized structures in which money and clicks promote what information is easily found can have damaging results on children and society. For instance, a year-long analysis by Noble (2018) showed that simply looking up information using the term “black girls” on Google brought up information that primarily focused on pornography. According to this study, if a young African-American girl used Google to look up images or websites related to her identity, she would see imagery of black women's bodies as objects for sexual desire. While not intentionally designed to cause harm, these systems use algorithms based on commercially motivated datasets that normalize one viewpoint and approach. Certain products or information tied to paying customers are likely going to be presented first. This begs the following question: how would a child bypass this information using a voice only interface? With traditional screens, the user can scroll past links without having to pay close attention to content of the advertisement. Therefore, it will be important to identify (a) the incentive structure that is used to build the AI system, (b) the types of information being used in the model, and (c) how that will be presented to children.

### Promoting Community Involvement

Finally, the communities and cultures that these conversations are used to train an AI have implications for not only children in the present but future generations of child users. As a persistent feature in society, AI systems can reify language and cultural conventions that can be integrated into the next generation of media and technology. Encouragingly, companies have made positive shifts toward diversifying books, television, and movies. However, that cannot be the stopping point for building AI-driven systems; inevitably, there must be community contribution and buy-in from actual children and families. Research has shown that socially connecting with media figures is important, particularly for marginalized youth who lack representation in their own environment, such as from the LBGTQIA+ community (Bond, 2018). Future conversational agents could include more gender-inclusive options instead of the default “her” or “him” with a range of tenor, tone, and even regional dialect. Finally, there will need to be a feedback structure that reengages communities as the systems and the world evolves. One possible solution could be to add a feature in which families could opt into providing feedback directly to the conversational agent on its performance. These types of changes can be made incrementally, but these small shifts can be powerful.

### Conclusion

Conversational agents powered by AI offer many applications and opportunities for children through social interactions. These tools must be carefully built and designed to meet children's developmental needs, and this can be achieved by using data based on how they think and behave. Technology design must be focused on the population it aims to serve, not on an imagined ideal user. Children's interactions with AI systems not only influence their actions and thoughts today but have implications on how they will interact with other people into the future.

## Data Availability Statement

The original contributions presented in the study are included in the article/supplementary material, further inquiries can be directed to the corresponding author/s.

## Author Contributions

JB developed the focus of manuscript and led the writing. BP and DG contributed to writing and editing sections of the manuscript. All authors contributed to the article and approved the submitted version.

## Conflict of Interest

The authors declare that the research was conducted in the absence of any commercial or financial relationships that could be construed as a potential conflict of interest.
